# Nicotinic receptors on rat alveolar macrophages dampen ATP-induced increase in cytosolic calcium concentration

**DOI:** 10.1186/1465-9921-11-133

**Published:** 2010-09-29

**Authors:** Zbigniew Mikulski, Petra Hartmann, Gitte Jositsch, Zbigniew Zasłona, Katrin S Lips, Uwe Pfeil, Hjalmar Kurzen, Jürgen Lohmeyer, Wolfgang G Clauss, Veronika Grau, Martin Fronius, Wolfgang Kummer

**Affiliations:** 1 Institute for Anatomy and Cell Biology, University of Giessen Lung Center, Justus-Liebig-University Giessen, Aulweg 123, D-35385 Giessen, Germany; 2 Institute of Animal Physiology, Justus-Liebig-University Giessen, Wartweg 95, D-35392, Giessen, Germany; 3 Division of Pulmonary and Critical Care Medicine, Department of Internal Medicine, University of Giessen Lung Center, Justus-Liebig-University Giessen, Klinikstr. 36, D-35392 Giessen, Germany; 4 Laboratory of Experimental Trauma Surgery, Department of Trauma Surgery, Justus-Liebig-University Giessen, Kerkraderstr. 9, D-35394, Giessen, Germany; 5 Department of Dermatology, Venereology and Allergology, University Medical Center Mannheim, Ruprecht-Karls-University Heidelberg, Theodor-Kutzer-Ufer 1-3, D-68135 Mannheim, Germany; 6 Laboratory of Experimental Surgery, Department of General and Thoracic Surgery, University of Giessen Lung Center, Justus-Liebig-University Giessen, Rudolf Buchheim Str. 7, D-35385 Giessen, Germany

## Abstract

**Background:**

Nicotinic acetylcholine receptors (nAChR) have been identified on a variety of cells of the immune system and are generally considered to trigger anti-inflammatory events. In the present study, we determine the nAChR inventory of rat alveolar macrophages (AM), and investigate the cellular events evoked by stimulation with nicotine.

**Methods:**

Rat AM were isolated freshly by bronchoalveolar lavage. The expression of nAChR subunits was analyzed by RT-PCR, immunohistochemistry, and Western blotting. To evaluate function of nAChR subunits, electrophysiological recordings and measurements of intracellular calcium concentration ([Ca^2+^]_i_) were conducted.

**Results:**

Positive RT-PCR results were obtained for nAChR subunits α3, α5, α9, α10, β1, and β2, with most stable expression being noted for subunits α9, α10, β1, and β2. Notably, mRNA coding for subunit α7 which is proposed to convey the nicotinic anti-inflammatory response of macrophages from other sources than the lung was not detected. RT-PCR data were supported by immunohistochemistry on AM isolated by lavage, as well as in lung tissue sections and by Western blotting. Neither whole-cell patch clamp recordings nor measurements of [Ca^2+^]_i _revealed changes in membrane current in response to ACh and in [Ca^2+^]_i _in response to nicotine, respectively. However, nicotine (100 μM), given 2 min prior to ATP, significantly reduced the ATP-induced rise in [Ca^2+^]_i _by 30%. This effect was blocked by α-bungarotoxin and did not depend on the presence of extracellular calcium.

**Conclusions:**

Rat AM are equipped with modulatory nAChR with properties distinct from ionotropic nAChR mediating synaptic transmission in the nervous system. Their stimulation with nicotine dampens ATP-induced Ca^2+^-release from intracellular stores. Thus, the present study identifies the first acute receptor-mediated nicotinic effect on AM with anti-inflammatory potential.

## Background

Alveolar macrophages (AM) hold a key position in initiating pulmonary inflammatory responses by secreting tumor necrosis factor α (TNFα) and several additional cytokines and chemokines. It has been demonstrated that TNFα production and release from peritoneal macrophages can be largely inhibited by neurally released ACh thereby attenuating systemic inflammatory responses. This physiological mechanism has been termed "cholinergic anti-inflammatory pathway" [1]. Studies on monocyte-derived human macrophages and on nicotinic acetylcholine receptor (nAChR) deficient mouse strains revealed that the nAChR α7 subunit is essential for this anti-inflammatory pathway [2]. It has been demonstrated that stimulation of mouse peritoneal macrophages with nicotine is associated with activation of the Jak2-STAT3 signaling pathway and with inhibition of the release of pro-inflammatory cytokines and chemokines [3]. Several lines of evidence show that stimulation of the cholinergic anti-inflammatory pathway and application of nicotinic agonists can be beneficial in experimental endotoxemia and sepsis [1-3]. The α7 subunit is one of 9 different known ligand-binding α subunits (α1-α7 and α9-α10) that assemble to homo- or heteropentamers, partially with additional participation of β subunits, to form a functional nAChR. All these receptors are ligand-gated cation channels, and they are distinct from each other with respect to ligand affinity and to preference for mono- or divalent cations [4]. There is growing evidence that neuronal-type ion channels are not formed by nAChR subunits in cells of the immune system [5-7].

In view of the natural occurrence of nAChR ligands in the alveolar compartment (e.g. choline) and of the clinical relevance of nicotine contained within cigarette smoke, the potential presence of a cholinergic anti-inflammatory pathway in the lung deserves high attention. Indeed, nAChR agonists reduce acid- and gram-negative sepsis-induced acute lung injury in mice and rats [8,9] and tumour necrosis factor-α (TNF-α) release into the lung compartment after intrapulmonary delivery of LPS in mice [10]. Here, we hypothesized that cholinergic anti-inflammation is operative through modulation of AM function. We established an inventory of nAChR subunit expression in rat AM by RT-PCR and immunohistochemistry. Whole-cell patch-clamp measurements were conducted to investigate whether classical, ion-conducting nAChR are operative in AM. The effect of nicotine upon macrophage stimulation with ATP, a "host tissue damage" or "danger signal" [11], was investigated by the method of real-time imaging for cytosolic Ca^2+ ^responses. We demonstrate that there is a nicotinic anti-inflammatory pathway operative in rat AM. The receptor subtypes involved and intracellular signaling pathways, as identified so far, differ from that known from the nervous system. Potentially, this allows for selective pharmacological intervention and therapeutic use.

## Methods

### Alveolar macrophage isolation

Female Wistar rats (8-10 weeks old) were obtained from the local animal breeding facility (Institute of Physiology, Justus-Liebig-University, Giessen, Germany) and kept under conventional conditions. Wild type C57BL6N specific-pathogen free (SPF) mice were purchased from Charles River (Sulzfeld, Germany). Mice deficient for the α7 nAChR subunit were obtained from Jackson Laboratory (Bar Harbor, USA) and bred in SPF conditions by the local animal breeding facility using heterozygotes as breeders. Male and female mice were used throughout the study between 8 and 12 weeks of age. All animals were kept with free access to food and water. Animal care and animal experiments were performed following the current version of the German Law on the Protection of Animals as well as the NIH "principles of laboratory animal care".

Animals were killed by inhalation of an overdose of isoflurane (Abbott, Wiesbaden, Germany). For isolation of rat AM, the lung was carefully removed, cannulated via the trachea, and bronchoalveolar lavage (BAL) was performed using 10 × 5 ml ice-cold PBS containing (in mM): KCl 2.68, KH_2_PO_4 _1.47, NaCl 136.89, Na_2_HPO_4 _8.10 (pH 7.3) (PAA, Pasching, Austria). Mouse AM were isolated according to previously described protocols [12]. The lavage fluid was centrifuged at 400 × g for 5 min at 4°C, and the pellet was resuspended in PBS or DMEM/F12 GlutaMax-I medium (Invitrogen, Karlsruhe, Germany). BAL cells were monitored by microscopy, and preparations containing erythrocytes were discarded. Isolated macrophages were used for subsequent analysis by RT-PCR, immunocytochemistry, Western blotting, electrophysiological recordings and measurements of intracellular calcium concentration ([Ca^2+^]_i_).

### RT-PCR

Total RNA was isolated from BAL cells (n = 7 rats and n = 5 C57BL6N mice) using RNeasy Mini Kit (Qiagen, Hilden, Germany). Genomic DNA contaminations were removed by DNase I digestion for 15 min at 37°C, 1 U/reaction (Invitrogen). cDNA synthesis was performed with iScript (Bio-Rad, Munich, Germany) or SuperScript II (Invitrogen) kits using 1 μg total RNA. The cDNAs were amplified with the subunit specific primer pairs spanning at least one intron (Table. 1 and 2). Hypoxanthine guanine phosphoribosyl transferase 1 (HPRT1) and β-microglobulin primers were used to monitor cDNA integrity for rat and mouse samples, respectively. For amplification, 2.5 μl buffer II (100 mM Tris-HCl, 500 mM KCl, pH 8.3), 2 μl 15 mM MgCl_2_, 0.6 μl dNTP (10 mM each), 0.6 μl of each primer (10 μM), and 0.125 μl AmpliTaq Gold polymerase (5 U/μl, Applied Biosystems, Foster City, USA) were added to 1 μl of cDNA, and made up to a final volume of 25 μl with H_2_O. Cycling conditions were 10 min at 95°C, 40 cycles with 20 s at 94°C, 20 s at 60°C, 20 s at 72°C, and a final extension at 72°C for 7 min. PCR products were separated by electrophoresis on a 1.5% agarose gel in Tris-acetate-EDTA buffer. Sequencing of PCR products was done by MWG Biotech (Ebersbach, Germany). Positive controls for primers detecting rat subunits α1 and β1 were done using reversely transcribed total RNA from rat muscle tissue. Rat lung was used as a positive control for subunits β2-β4. For other rat primer pairs, rat DRG were used as a positive control. Mouse lung was used as a positive control for all primers detecting mouse nAChR subunits. Negative controls were performed by omitting the reverse transcription step or using water instead of cDNA template.

**Table 1:** Rat primer sequences used in the study.

Target	Sequence		Length	**Accession No**.
nAChR α1	for.	AGCTCACCGCTGTCCTCCT	171 bp	[GenBank:NM_024485]
	rev.	GGATCAGTTGCAGTCCCACA		
nAChR α2	for.	CGCGTCCCTTCAGAGATGAT	114 bp	[GenBank:L31622]
	rev.	CACAGTGCCCGTGAAGAA		
nAChR α3	for.	CCTCCCTGTCTATCGGGTCT	161 bp	[GenBank:X03440]
	rev.	GCCGGATGATCTCGTTGTAA		
nAChR α4	for.	GGACCCTGGTGACTACGAGA	137 bp	[GenBank:NM_024354]
	rev.	CATAGAACAGGTGGGCCTTG		
nAChR α5	for.	TGGAACACCTGAGCGACAAG	284 bp	[GenBank:NM_017078]
	rev.	CGTGACAGTGCCGTTGTACC		
nAChR α6	for.	TGGTGTTAAGGACCCCAAAA	142 bp	[GenBank:NM_057184]
	rev.	GCTGCTGGCTTAACCTCTTG		
nAChR α7	for.	ACATTGACGTTCGCTGGTTC	235 bp	[GenBank:L31619]
	rev.	CTACGGCGCATGGTTACTGT		
nAChR α9	for.	CGTGGGATCGAGACCAGTAT	142 bp	[GenBank:AY574257]
	rev.	TCATATCGCAGCACCACATT		
nAChR α10	for.	GTGCCACTCATCGGAAAGTA	107 bp	[GenBank:NM_022639]
	rev.	TGTGCATTAGGGCCACAGTA		
nAChR β1	for.	TCCTAAGCGTGGTGGTCCTC	151 bp	[GenBank:NM_01258]
	rev.	TGTGGTTCGGGTAGTTGGTC		
nAChR β2	for.	AGCCTTCTTTGGCTGTGCTC	116 bp	[GenBank:NM_019297]
	rev.	GAGCCGTTAGTAGCTGGACGA		
nAChR β3	for.	CACTCTGCGCTTGAAAGGAA	196 bp	[GenBank:NM_133597]
	rev.	GCGGACCCATTTCTGGTAAC		
nAChR β4	for.	CACTCGCGGTTCCATTGTAG	159 bp	[GenBank:NM_052806]
	rev.	CGGGTTTTGTTCAGGAGGTC		
P2Y1	for.	AGGAAAGCTTCCAGGAGGAG	203 bp	[GenBank:NM_012800]
	rev.	GGCCAATAGAATGTTGCTTCTT		
P2Y2	for.	CATGCGAGTGAAGAACTGGA	209 bp	[GenBank:NM_017255]
	rev.	GGCAGCAGCACATACTTGAA		
P2Y4	for.	AGTCCCTGGGCTGGACTAAG	267 bp	[GenBank:NM_031680]
	rev.	GTGTCTGACAATGCCAGGTG		
HPRT1	for.	TCCCAGCGTCGTGATTAGTG	225 bp	[GenBank:NM_012583]
	rev.	TCCAGCAGGTCAGCAAAGAA		

Sequences for forward (for.) and reverse (rev.) primers are given in 5'→3' order.

**Table 2:** Mouse primer sequences used in the study.

Target	Sequence		Length	**Accession No**.
nAChR α7	for	ACAATACTTCGCCAGCACCA	144 bp	[GenBank:AF225980]
	rev	AAACCATGCACACCAGTTCA		
nAChR α9	for	CAATGCTCTGCGTCCAGTAG	209 bp	[GenBank:XM_132045]
	rev	ACACCAGATCGCTGGGAATC		
nAChR α10	for	TCTGCTCCTGCTCTTTCTCC	208 bp	[GenBank:XM_89067]
	rev	CCACAGGTACAAGGTCAGCA		
nAChR β2	for	GAGTGTGAGGGAGGATTGGA	139 bp	[GenBank:AY574268]
	rev	TCGTGGCAGTGTAGTTCTGG		
nAChR β4	for	CAGCCCATCCAACCTCTATG	156 bp	[GenBank:NM_148944]
	rev	CTGACGCCCTCTAATGCTTC		
β-MG	for	ACCCTGGTCTTTCTGGTGCT	150 bp	[GenBank:NM_009735]
	rev	AATGTGAGGCGGGTGGAA		

Differences were noted for two methods used to prepare cDNA. Successful detection of rat α9 subunit mRNA required reverse transcription with Superscript II system. Amplification of rat α9 subunit mRNA from BAL cDNA generated with iScript enzyme was not successful. This may be due to different priming strategies (oligo(dT) and blend of oligo(dT) + random hexamer primers, respectively) or to reduced RNAse H activity in SuperScriptII enzyme, which enables more efficient cDNA synthesis [13].

### Immunofluorescence

Lavaged rat cells (n = 10 animals) were plated on polystyrene 8-well culture slides (BD Biosciences, Erembodegem, Belgium) in DMEM/F12 supplemented with penicillin (100 U/ml) and streptomycin (0.1 mg/ml). Cells were allowed to attach for 2 h, and then fixed in acetone (-20°C, 10 min) or isopropanol (+4°C, 10 min) and air-dried for 1 h.

Shock-frozen rat lung specimens were prepared as described previously [14]. Cryostat sections were cut at 10 μm thickness, fixed as above and subjected to indirect immunofluorescence using antisera directed against nAChR subunits and monoclonal antibody ED1, directed to a CD68-like antigen expressed by rat AM [15] (Serotec, Düsseldorf, Germany) (Table. 3). Briefly, unspecific binding sites were saturated with 50% normal horse serum in PBS for 1 h, followed by overnight incubation with the primary antibody, washing (3 × 10 min) and application of secondary antibody for 1 h. Slides were washed, fixed in buffered 4% paraformaldehyde, coverslipped in carbonate-buffered glycerol (pH 8.6) and examined with a Zeiss Axioplan 2 microscope (Zeiss, Jena, Germany) and sequential confocal laser scanning microscope (CLSM, TCS SP2, Leica, Bensheim, Germany) using argon and HeNe lasers equipped with appropriate filter sets. Secondary antisera were Cy3-coupled donkey anti-rabbit IgG (1:2000 in PBS, Chemicon, Hofheim, Germany), Cy3-coupled donkey anti-guinea pig IgG (1:800 in PBS, Dianova, Hamburg, Germany), fluorescein-isothiocyanate-conjugated donkey anti-mouse Ig (1:400, Dianova), and Texas Red^®^-conjugated donkey anti-guinea pig Ig (1:100, Dianova). Positive controls were run on shock-frozen and acetone-fixed DRG sections. The specificity of the immunolabeling was validated by omission of the primary antibody or preincubation with the corresponding antigen. Preabsorption was done by mixing antibodies with peptide used for immunization (14-24 μg of peptide per 100 μl of antibody solution) for 1 h before application on slides. Peptides were obtained from the same source as the antibodies (Table. 3).

**Table 3:** Antibodies used in the study.

Target	Immunogen	Host	Dilution	Source
nAChR α3	Synthetic peptide (aa 466-474 of human sequence)^a^	Rabbit	1:1600	Acris
nAChR α4	Synthetic peptide (620-627, human)^a^	Rabbit	1:800	Acris
nAChR α5	Synthetic peptide (460-468, human)^a^	Rabbit	1:1600	Acris
nAChR α7	Synthetic peptide (493-502, human)^a^	Rabbit	1:1000	Acris
nAChR α7	Native and denatured α7 subunit (380-400, chicken) and denatured α7 subunit from rat	Mouse, monoclonal, clone mAb 306	1:750	Sigma-Aldrich
nAChR α9	Synthetic peptides (81-97 and 115-128, rat)	Guinea-pig	1:1000	[44]
nAChR α10	Synthetic peptide (404-418, rat)^a^	Rabbit	1:2000	[17]
nAChR β2	Synthetic peptide (493-502, human)^a^	Rabbit	1:1600	Acris
nAChR β3	Synthetic peptide (450-458, human)^a^	Rabbit	1:800	Acris
nAChR β4	Synthetic peptide (490-498, human)^a^	Rabbit	1:3200	Acris
CD68-like	Rat spleen cells	Mouse, monoclonal, clone ED1	1:800	Serotec
pSTAT3 Tyr705	Synthetic phospho-peptide residues surrounding Tyr705 of mouse Stat3	Rabbit, monoclonal, clone D3A7	1:1000	Cell Signaling
pSTAT3 Ser727	Synthetic phospho-peptide residues surrounding Ser727 of mouse Stat3	Rabbit, monoclonal	1:1000	Cell Signaling
STAT3	Synthetic peptide corresponding to the sequence of mouse Stat3	Rabbit, monoclonal	1:1000	Cell Signaling

Origin and regions of nAChR subunits sequences used for generation of the antibodies are provided in brackets. ^a^Peptides available for preabsorption.

### SDS-PAGE and immunoblotting

#### nAChR α7 and α10 subunits detection

Snap-frozen rat BAL cells, rat brain and skin samples were homogenized and boiled in Laemmli's sample buffer [16] containing Complete^® ^protease inhibitor cocktail (Roche, Mannheim, Germany). SDS-PAGE was carried out using 15% polyacrylamide gels according to Laemmli et al. [16]. Samples (5 × 10^4 ^cells) and Rainbow™ colored molecular mass markers (Amersham Pharmacia Biotech, Freiburg, Germany) were separated on the same gel. Proteins were transferred electrophoretically onto Immobilon™-P PVDF membranes (Millipore, Bedford, USA) using a blotting buffer consisting of 25 mM Tris, 192 mM glycine, 20% methanol and 0.05% SDS. Membranes were pre-incubated with PBS containing 10% Rotiblock (Roth, Karlsruhe, Germany) solution for 1 h. Primary mouse-anti-nAChR α7 (1:1000, Sigma-Aldrich, Taufkirchen, Germany) antibodies were diluted in blocking solution and incubated with membranes overnight at 4°C. For the detection of α10 subunits, membranes were pre-incubated with PBS containing 5% non-fat milk powder (Roth) and guinea pig-anti-nAChR α10 (1:4000, [17]) antibodies were used. Blots were washed in PBS, 0.05% Tween 20 and bound primary antibodies were detected by horseradish peroxidase-conjugated immunoglobulins (DAKO, Hamburg, Germany) in PBS, 2% low fat milk, 0.05% Tween 20 (TPBS). Secondary antisera were rabbit anti-mouse IgG (1:5000 in TPBS + 1% normal rat serum) and rabbit-anti-guinea pig IgG (1:5000 in TPBS + 2% low fat milk). Peroxidase activity was visualized by SuperSignal^® ^West Pico Chemiluminescent Substrate (Pierce, Rockford, IL, USA) using the Kodak Scientific Imaging Film X-OMAT™ LS (Eastman Kodak, Rochester, NY, USA). Gels and blots were documented and densitometrically analyzed using a digital gel documentation system (Biozym, Hessisch Oldendorf, Germany).

#### STAT3 phosphorylation

Lavaged rat cells were plated on a 24 well plate (Becton Dickinson, USA) at 3.5 × 10^5 ^cells/well in RPMI 1640 medium supplemented with L-glutamine, penicillin (100 U/ml) and streptomycin (0.1 mg/ml). Cells were allowed to attach for 2 h and subsequently were stimulated with nicotine (Sigma-Aldrich) at 10^-4 ^M, 10^-5 ^M, and 10^-6 ^M or GM-CSF (R&D Systems, Minneapolis, MN) at 100 ng/ml for indicated time intervals. Cells were washed twice with cold PBS and lysed with lysis buffer containing 20 mM Tris (pH 7.5), 150 mM NaCl, 1 mM EDTA (pH 8.0), 1 mM EGTA (pH 8.0), 0.5% NP-40, 2 mM sodium orthovanadate (pH 10.0), and Complete^® ^protease inhibitor cocktail (Roche). The lysates were kept on ice for 30 min, followed by centrifugation for 15 min at 13,000 rpm at 4°C, and subsequent protein concentration measurement was assessed by Bradford Assay as suggested by the manufacturer (Bio-Rad). Proteins were loaded on a gel in a total amount of 10 μg, separated by electrophoresis on 10% SDS-PAGE, and transferred to polyvinylidene difluoride membranes (Amersham GE Healthcare, Little Chalfont, Buckinghamshire, UK). Membranes were incubated in blocking buffer (5% non-fat milk in PBS, 0.05% Tween 20) at room temperature for 1 h, and then overnight at 4°C with primary antibodies recognizing total and phosphorylated STAT3 (1:1000, Cell Signaling Technology, Beverly, MA). Blots were washed three times for 15 min, and incubated with horseradish peroxidase-conjugated anti-rabbit IgG (1:3500, Pierce, Rockford, IL). Enhanced chemiluminescence system was used to visualize immune complexes (Amersham GE Healthcare, Little Chalfont, Buckinghamshire, UK).

### Electrophysiological recording

For whole cell patch-clamp recordings, 200 μl cell suspension obtained from rat BAL was placed in recording dishes (Nunc, Roskilde, Denmark), incubated for 1-3 h at 37°C and 5% CO_2 _in DMEM/F12 medium to allow the cells to attach. PC12 cells (rat adrenal pheochromocytoma cells) were obtained from German Collection of Microorganisms and Cell Cultures (Braunschweig, Germany) and maintained at 37°C and 5% CO_2 _in RPMI 1640 medium supplemented with 10% horse serum (PAA), 5% FCS, 2 mM L-glutamine, penicillin (100 U/ml), streptomycin (0.1 mg/ml) and used as described above.

For recordings, the medium was carefully removed, cells were washed 2-3 times, covered with bath solution containing (in mM): NaCl 120, KCl 5.4, CaCl_2 _2, MgCl_2 _1, Hepes 10, D-glucose 25, pH 7.5, and the dish was placed under a microscope (Axiovert, Göttingen, Germany).

Borosilicate glass capillaries (Hilgenberg, Malsfeld, Germany) with an outer diameter of 1.6 mm were pulled to recording pipettes by a vertical puller (Narishige, Tokyo, Japan). The tips of the pipettes were fire-polished using a microforge (List-Medical, Darmstadt, Germany) and had resistances between 5-10 MΩ when filled with the pipette solution containing (in mM): KCl 120, CaCl_2 _1, MgCl_2 _2, Hepes 10, EGTA 11, D-glucose 20, pH 7.3. The junction potential under these conditions (bath and pipette solution) was 3.4 mV although the membrane voltage was not corrected with respect to this junction potential.

The whole cell configuration was mainly obtained by suction and in some cases voltage pulse was additionally applied. Transmembrane currents were recorded at holding potentials of -60 mV in the absence of, as well as after ACh application into the bath. The agonist was applied via a pipette to the bath to reach a final concentration of 100 μM. In some experiments, a VM-4 micro-perfusion system was used for drug compound application (ALA Scientific Instruments, Westbury, USA). The measured signals were amplified by an EPC-9 patch-clamp amplifier (HEKA, Lambrecht/Pfalz, Germany), which was connected via an ITC-16 interface to a personal computer. For continuous recordings, the signals were filtered with 300 Hz and acquired at 3 kHz using the Pulse 8.77 software (HEKA). Current/voltage relationships signals were filtered with 3.33 kHz and acquired at 10 kHz. Data ware analyzed and prepared using PulseFit (HEKA) and Igor (Wavemetrics, Lake Oswego, USA). All recordings were performed at room temperature.

### Intracellular calcium concentration measurements

Recordings of [Ca^2+^]_i _were performed after 3-8 h in primary culture (n = 3 animals and 10-12 coverslips for each experimental setup). Measurements were done in Hepes buffer containing (in mM): KCl 5.6, NaCl 136.4, MgCl_2 _1, CaCl_2 _2.2, D-glucose 11, Hepes 10. In some experiments, CaCl_2 _was omitted from the buffer composition. Cells were loaded for 30 min with 3.3 μM fura-2 AM (Invitrogen) and washed 3 × 10 min. Fura-2 was excited at 340 and 380 nm wavelengths (λ), and fluorescence was collected at λ > 420 nm. The fluorescence intensity ratio of 340/380 nm was recorded. Cells were exposed to nicotine (10^-6^-10^-4 ^M, Sigma-Aldrich) or epibatidine (10^-6 ^M, Sigma-Aldrich). Controls were performed with vehicle treatment. Two min after administration of nicotine or epibatidine, cells were stimulated with ATP (10^-4 ^M, Sigma-Aldrich). In some experiments cells were exposed to nicotine in the presence of the nAChR α7 and α9/α10 blocker α-bungarotoxin (10^-7 ^M, Sigma-Aldrich). Cells that did not respond to ATP by at least 5% change in [Ca^2+^]_i _were excluded from further analysis. Viability of the cells was monitored after measurements with Trypan Blue exclusion. Ratio values were normalized to 100% at the beginning of recording. Curves were plotted from recordings done in preparations from n = 3 animals. Data are shown as mean ± SEM.

### Statistical analysis

Data in the figures and text are expressed as mean ± SEM. Non-parametric rank based Kruskal-Wallis test was used to compare multiple groups, and if significant differences were detected, it was followed by Mann-Whitney test to compare between two experimental groups. Tests were performed using SPSS software (SPSS software, Munich, Germany). P ≤ 0.05 was considered significant and P ≤ 0.01 as highly significant.

## Results

### Rat alveolar macrophages constitutively express nAChR subunits α9, α10 and β2, but not α7

**Figure 1:**
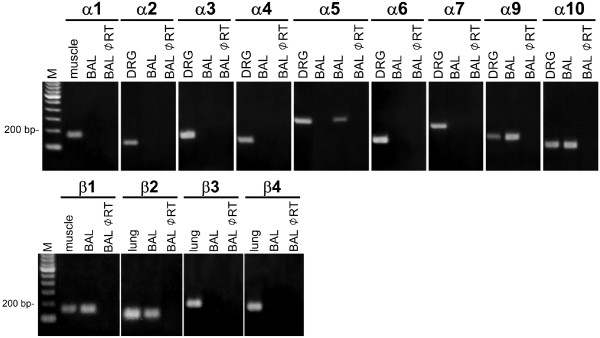
**RT-PCR analysis of nAChR subunits in BAL cells, which consistently express mRNA coding for α9, α10, β1 and β2 subunits**. Subunit α5 shows an interindividual expression pattern. The mRNAs coding for α1, α4, α6, α7 and β3 subunits are absent. Positive controls were run on DRG (α2-α10), lung (β2-4), and skeletal muscle (α1 and β1). Negative controls were done without RT.

RT-PCR analysis of mRNA isolated from rat BAL cells revealed expression of nAChR subunits α2, α3, α5, α9, α10, β1, and β2. Products corresponding to the α4, α6, α7, and β3 subunits were never found in BAL cells (Fig. 1), although they were easily detectable in DRG and lung homogenate (α7 and β3 subunits). The mRNAs of subunits α9, α10, β1, and β2 were consistently expressed. Subunits α2, α3, and β4 were detected in 1 out of 9 preparations. Subunit α5 was present in 3 out of 9 preparations. The identity of amplified products was confirmed by sequencing, and control runs without template were negative.

**Figure 2:**
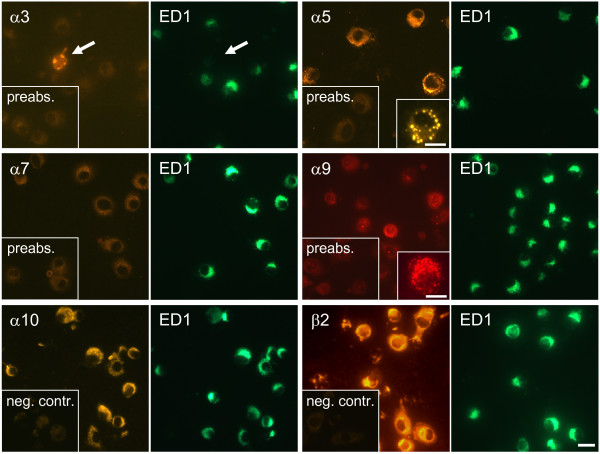
**Double-labeling immunofluorescence demonstrating the presence of the nAChR subunits α5, α9, α10 and β2 in ED1-positive BAL cells**. A punctate fluorescence pattern for the α5 and α9 nAChR subunits was found in a subset of AM *(inserts)*. Negative results were noted for α3 and α7 subunits in ED1-positive cells, despite occasional occurrence of α3 subunit immunoreactive ED1-negative cells *(arrow)*. Bar: 20 μm, 10 μm in inserts.

**Figure 3:**
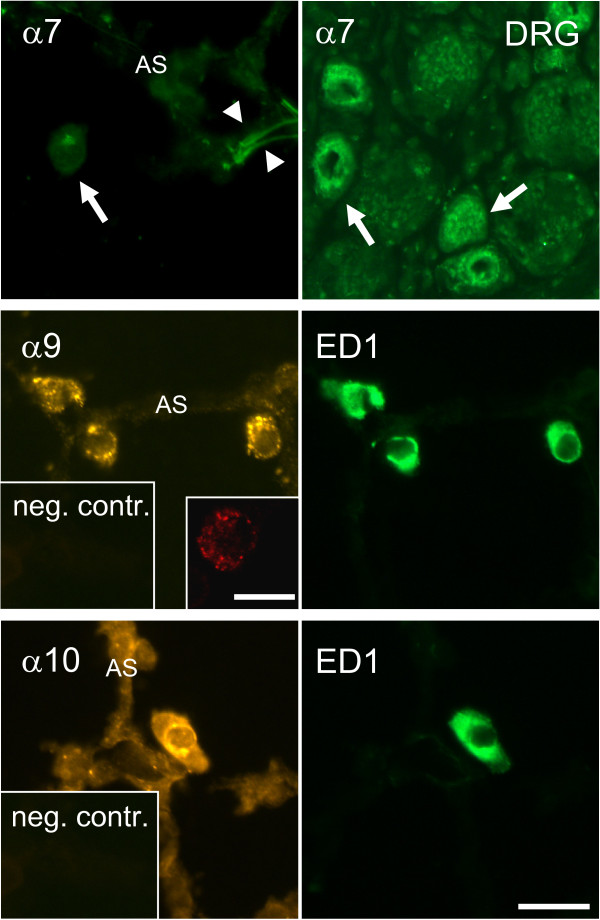
**Immunohistochemistry on rat lung sections and DRG (positive control)**. The mAb 306 directed against the α7 subunit fails to label AM *(arrow) *but it stains nerve cell bodies *(arrows) *in DRG sections serving as a positive control. *Arrowheads: *Elastin autofluorescence in alveolus. The antibodies to α9 and α10 subunits label ED1-positive AM in alveoli. The *insert *demonstrates punctate α9 subunit immunoreactivity in a CLSM optical section. *AS *= alveolar septum. Bar: 20 μm.

In indirect double-labeling immunofluorescence, the vast majority of rat BAL cells showed strong staining with ED1 antibody, an AM marker. ED1-positive cells were immunoreactive for α9, α10, and β2 nAChR subunits, and in 50% of BAL preparations (5 out of 10 samples) for the α5 subunit as well. Preabsorption with the synthetic peptides used for immunization resulted in absence of immunolabeling (Fig. 2). Staining was predominantly intracellular, localized near the nucleus, except for α5 and α9 subunit-immunoreactivity that exhibited a punctate surface distribution in a subset of AM (Fig. 2, inserts). The rabbit polyclonal antibody to α7 subunit faintly stained AM, but preabsorption with corresponding peptide gave the same staining pattern. The monoclonal antibody to the α7 nAChR subunit (mAb 306) did not show any labeling of AM. Positive controls were run on DRG sections, demonstrating labeling of neuronal cell bodies with mAb 306 (Fig. 3). There was no specific labeling for subunits α3, α4, β3, and β4 in ED1-positive cells (Fig. 2 and data not shown). Similar staining patterns with antisera directed against nAChR subunits were observed in rat lung sections (Fig. 3). Here, the antiserum against the α9 nAChR subunit showed marked punctuate membrane staining of AM (Fig. 3, *insert*).

**Figure 4:**
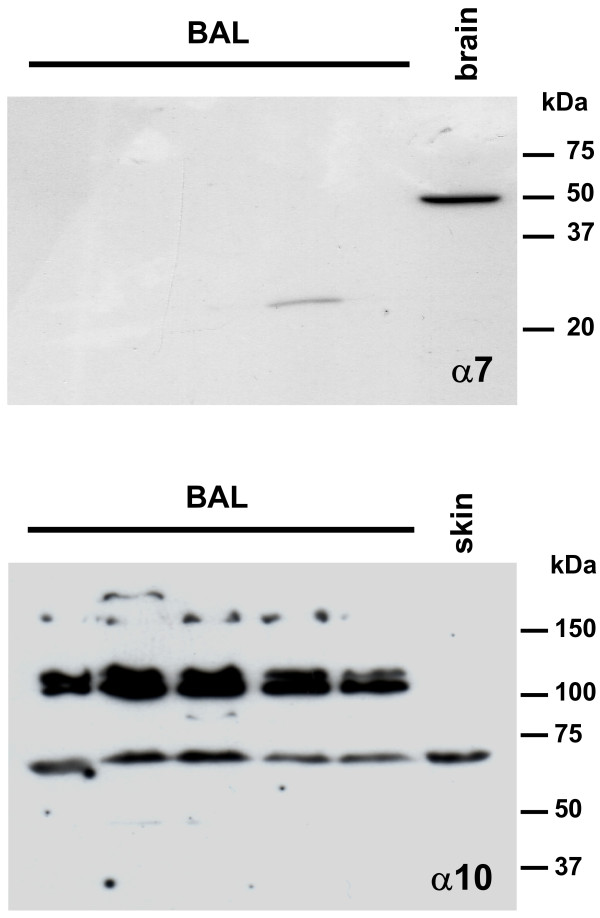
**Immunoblots**. No α7 subunit-immunolabeling is present in BAL samples, while the antibody mAb 306 recognizes a single 50 kDa protein band in protein extracts from rat brain. Affinity purified polyclonal antibodies to α10 nAChR label a protein band at 67 kDa in BAL cells and rat skin samples. In BAL cells, additional bands at 110-120 kDa are immunolabeled.

Western blotting supported the immunohistochemical findings in that the mAb 306 failed to detect the α7 subunit in protein preparations from rat BAL cells while it recognized a ~50 kDa band in protein extracts from rat brain (Fig. 4). In Western blots of rat skin homogenates serving as a positive control, a single 67-kDa protein was recognized utilizing a previously characterized antiserum directed against the α10 nAChR subunit [12]. As shown in Fig. 4, this band plus additional bands at 110-130 kDa were immunolabeled in protein preparations from BAL cells.

### Acetylcholine has no effect on AM cell membrane conductivity

**Figure 5:**
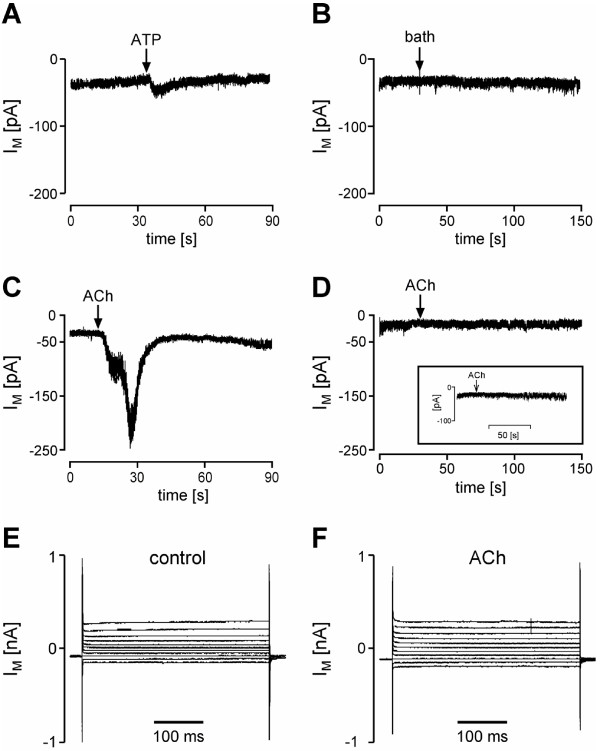
**Patch clamp recordings on AM (A, B, D-F) and on PC12 cells (C)**. (A) Representative whole cell recording depicting the measured membrane current (I_M_) at -60 mV in absence of and following ATP application (2 × 10^-4 ^M, indicated by arrow). Agonist was applied directly to the bath via a pipette. ATP activates transient inward currents. (B) Control recording where bath solution was applied instead of agonist. (C) Effect of ACh (10^-4 ^M) on PC12 cells. ACh induces strong inward currents. (D) Application of ACh to the bath solution has no effect on membrane currents in AM. Insert: The use of a perfusion system also did not induce any significant changes of the membrane current in response to ACh. (E, F) Current-voltage relationship recorded in the absence of (control, E) and immediately after ACh application (F). In these recordings, again no changes in membrane current were detected.

Recordings were performed at -60 mV holding potential. ATP (2 × 10^-4 ^M) induced currents in approximately 50% of the recorded rat AM (Fig. 5A). Notably, no changes in membrane currents were detected when ACh (10^-4 ^M) was added to the bath (Fig. 5D, n = 15). In a separate set of recordings, ACh was applied via a perfusion system, again without triggering changes in transmembrane current (I_M_) (see insert of figure 5D). In contrast, PC12 cells readily responded to ACh application by an increased inward current (Fig. 5C).

For further characterization of AM responses, I/V curves were recorded by applying voltage steps of 20 mV from -100 to 100 mV, starting from a holding potential of -60 mV. I/V-relationships were also recorded in the absence (Fig. 5F) and presence of ACh (Fig. 5E). Again, no changes of I_M _were evoked by ACh application.

### ATP-triggered increase in calcium derives from intracellular stores

**Figure 6:**
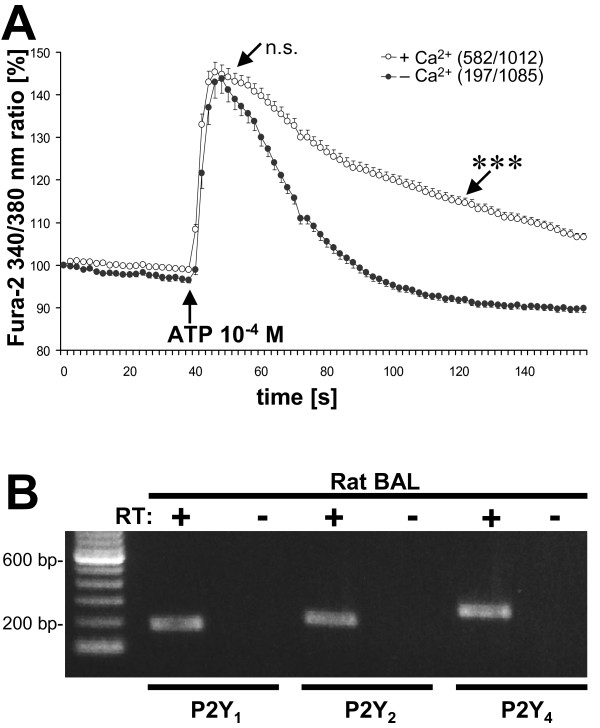
**Purinergic receptors on freshly isolated BAL cells**. ATP-induced transients in [Ca^2+^]_i _are mainly dependent on intracellular stores. (A) BAL cells were stimulated with ATP (10^-4 ^M) in the presence or absence of extracellular Ca^2+^. Sustained increase, but not initial rise in [Ca^2+^]_i _is dependent on extracellular Ca^2+^. Number of cells which reacted, and total number of measured cells are given in brackets. n.s. = not significant, ***P ≤ 0.001. (B) RT-PCR in rat BAL cells shows expression of P2Y_1_, P2Y_2 _and P2Y_4 _purinergic receptors.

In freshly isolated rat AM, ATP (10^-4 ^M) induced a rapid rise in [Ca^2+^]_i _followed by a slow decrease. Exclusion of calcium ions from the external solution had no effect on the amplitude of the ATP-induced initial [Ca^2+^]_i _rise (46% for +Ca^2+ ^and 47% for -Ca^2+ ^Hepes buffer). Macrophages exposed to extracellular Ca^2+ ^showed a sustained increase in [Ca^2+^]_i _whereas cells stimulated in Ca^2+^-free buffer showed only a transient rise without reaching a plateau phase (P ≤ 0.001) (Fig. 5A). The percentage of cells reacting to the ATP stimulus was decreased from 58% in calcium-containing to 18% in calcium-free buffer (582/1012 cells in +Ca^2+ ^vs 197/1085 cells in -Ca^2+ ^buffer). To better understand which receptors might be involved in ATP-induced [Ca^2+^]_i _rise we performed RT-PCR analysis and found P2Y_1_, P2Y_2 _and P2Y_4 _purinergic receptor mRNAs in AM (Fig. 6B).

### Nicotine modulates ATP-induced rise in intracellular [Ca^2+^]

**Figure 7:**
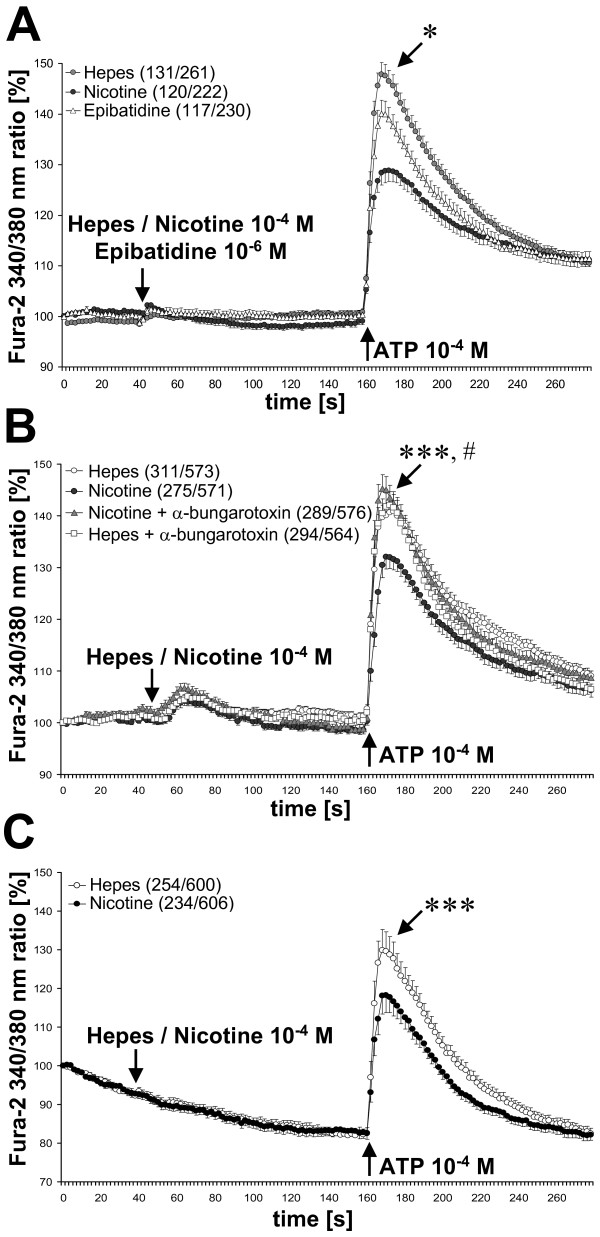
**Nicotine dampens ATP-induced increase in [Ca^2+^]_i_**. (A) BAL cells were stimulated with ATP (10^-4 ^M) in the presence or absence of nicotine (10^-4 ^M) or epibatidine (10^-6 ^M). * P ≤ 0.01 (nicotine compared to Hepes) (B) The nicotine-induced effect is blocked by pre-incubation with the nicotinic antagonist α-bungarotoxin (10^-7 ^M). ***P ≤ 0.001 (nicotine compared to α-bungarotoxin + nicotine) ^#^P ≤ 0.05 (Hepes compared to α-bungarotoxin + nicotine). (C) Nicotine-mediated effect on ATP-induced [Ca^2+^]_i _rise is not depended on extracellular calcium. BAL cells were treated with nicotine (10^-4 ^M) or with the vehicle in the presence of extracellular Ca^2+^. Immediately before measurements, cells were transferred to Ca^2+^-free buffer where a constant leakage of Ca^2+ ^from the cells can be observed. BAL cells were pre-treated with nicotine or with the vehicle, and then stimulated with ATP (10^-4 ^M). Number of cells which reacted, and total number of measured cells are given in brackets. ***P ≤ 0.001, *P ≤ 0.05.

We tested whether nicotine modulates [Ca^2+^]_i _levels. Half of the macrophage population (131/261 cells) exposed to ATP showed a transient rise in [Ca^2+^]_i _(increase by 47%). Application of nicotine (10^-6^, 10^-5^, 10^-4 ^M) or epibatidine (10^-6 ^M) had no direct effect on [Ca^2+^]_i_. Nicotine given 2 min prior to ATP reduced the ATP-induced calcium peak by 38% (P ≤ 0.006), while not changing the percentage of cells reacting to the ATP stimulus (54%, 120/222 cells). Epibatidine was neither effective in reducing ATP-induced transients nor it changed the percentage of cells reacting to the ATP (51%, 117/230 cells) (Fig. 7A).

In a separate set of experiments, we tested if the effect of nicotine can be blocked with the α1, α7 and α9/α9α10 nAChR antagonist α-bungarotoxin. This drug alone (10^-7 ^M) had no effect on ATP-induced calcium increase, when compared to vehicle control, but it abrogated the effects of nicotine (P ≤ 0.001). The transient rise in [Ca^2+^]_i _in cells treated with α-bungarotoxin together with nicotine was slightly increased compared to vehicle-treated cells (P ≤ 0.025) (Fig. 7B).

**Figure 8:**
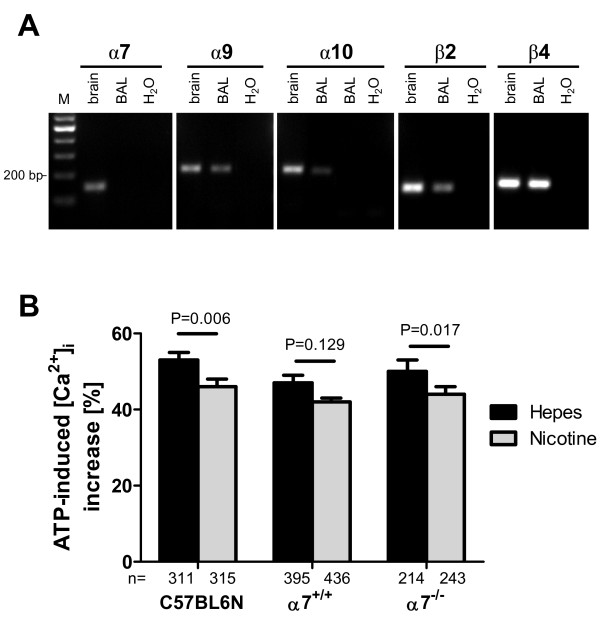
**Nicotinic receptors on mouse BAL cells**. (A) RT-PCR, agarose gel electrophoresis. Mouse BAL cells consistently express mRNAs coding for α9, β2 and β4 subunits. Subunit α10 shows an interindividual expression pattern. The mRNA for α7 subunit was not found in BAL preparations, although it was easily detectable in brain homogenate, serving as a positive control. M = DNA marker, H_2_O = water control (B) Ratiometric [Ca^2+^]_i _recordings. Mouse BAL cells isolated from C57BL6N, α7 nAChR knockout (α7^-/-^) and littermate control animals (α7^+/+^) were stimulated with ATP (10^-4 ^M) in the presence or absence (Hepes) of nicotine (10^-4 ^M). Peak increases shown as mean ± SEM; cells taken from 6-9 coverslips for each experimental setup, p-values calculated by Mann-Whitney test.

We used AM isolated from α7 nAChR-deficient mice to determine the role of this subunit in cholinergic modulation of ATP-induced Ca^2+ ^response. Cells isolated by BAL from C57BL6N mice expressed mRNAs corresponding to α9, β2 and β4 nAChR subunits (Fig. 8A). Messenger RNA for the α10 subunit was found in 3 out of 5 samples, while α7 nAChR subunit mRNA was never detected in BAL cells. Pretreatment with nicotine significantly attenuated the transient rise in [Ca^2+^]_i _triggered by ATP in BAL cells isolated from C57BL6N and α7 nAChR-deficient mice (Fig. 8B), albeit the reduction was much less pronounced than in rat cells.

### Nicotinic modulation is not dependent on extracellular calcium

Next we tested if the nicotine-mediated effect upon the ATP-induced [Ca^2+^]_i _rise is depended on extracellular calcium. Rat cells treated with nicotine (10^-4 ^M) 2 min before the ATP stimulus showed a decreased amplitude of the ATP-induced rise in [Ca^2+^]_i_. This was not affected by the absence of Ca^2+ ^in the external bath solution (Fig. 7B). The number of cells reacting to ATP was reduced when Ca^2+ ^was omitted in the external solution (18% for vehicle and 16% for nicotine treated cells) compared to Ca^2+^-supplemented medium (42% for vehicle and 39% for nicotine treated cells).

### Nicotine does not induce STAT-3 phosphorylation

**Figure 9:**
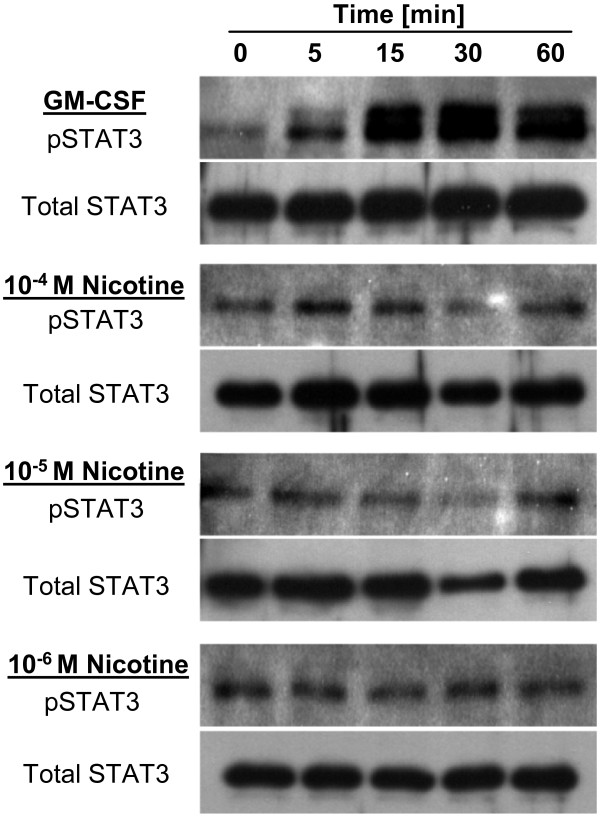
**Nicotine does not induce STAT-3 phosphorylation**. BAL cells were stimulated with nicotine at 10^-4 ^M, 10^-5 ^M, and 10^-6 ^M or GM-CSF at (100 ng/ml) for 0, 5, 15, 30 and 60 min, as indicated. Primary rabbit antibodies directed to phospo-STAT 3 (Tyr 705) or to total STAT 3 were used. Only GM-CSF induced phosphorylation of STAT3, as shown on the blot.

To test whether nicotinic stimulation of BAL cells triggers STAT-3 phosphorylation, as it has been reported for peritoneal macrophages [3], Western blot analysis was performed. Rat BAL cells were exposed to 10^-4^, 10^-5^, and 10^-6 ^M nicotine or to 100 ng/ml GM-CSF serving as a positive control. No visible STAT-3 phosphorylation on Tyr705 epitope was observed in nicotine treated samples 5, 15, 30 and 60 min after stimulation. Similar results were obtained with an antibody recognizing phosphorylation of Ser727 epitope (data not shown). In contrast to nicotine, GM-CSF caused profound STAT-3 phosphorylation as soon as 5 min after addition to the cells (Fig. 9).

## Discussion

This study is the first to demonstrate acute receptor-dependent, modulatory effects of nicotine on AM. The nAChR involved in this process differ from subtypes reported previously to be involved in "cholinergic anti-inflammatory pathways" outside the lung. Although the effects of nicotine are receptor mediated, these receptors do not form a classical ion channel known from neuronal cells.

Importantly, we detected neither mRNA nor protein of α7 nAChR in AM in contrast to easily detectable α7 subunit mRNA in sensory neurons, brain and in the whole lung homogenate. This is consistent with reported data on the lack of the expression of α7 nAChR in the murine AM cell line MH-S [18] and our previous work on expression of nAChR in freshly isolated murine AM [19]. In contrast, binding of the polyclonal nAChR α7 antibody H-320 to murine AM has been reported by Su et al. [8,9] and was also noted by our group in a previous study in the absence of α7 subunit mRNA detection [19]. This antibody, however, produces identical staining in immunohistochemistry and western blotting of the mouse brain, clearly demonstrating lack of specificity at least in the nervous system [20], so that these findings have to be considered with caution unless corresponding controls on mouse lungs from α7 nAChR^-/- ^mice have been successfully performed.

Still, there might be species differences and plasticity in receptor expression under pathological conditions, since a low level of basal expression of α7 nAChR subunit mRNA in AM isolated from healthy volunteers and an increase in AM isolated from smokers has been reported [21]. Also, α7 nAChR are essential for systemic cholinergic anti-inflammation since the beneficial effects of nicotine in endotoxemia are abrogated in α7 subunit gene-deficient mice [2]. Accordingly, two potent α7 nAChR agonists, GTS-21 and PNU-282987 [22,23], inhibit LPS-induced TNFα release and reduce acid-induced acute lung injury, respectively, in the mouse lung [8,10]. Their potency on the most prevalent nAChR subunits identified in our present study on AM, i.e. α9 and α10 nAChR that generally share many pharmacological properties with α7 nAChR [24], yet has not been determined. Without doubt, however, α7 nAChR is expressed in the lung as demonstrated by RT-PCR in this and previous studies [25,26]. Functional data show increases in acid-induced excess lung water and vascular permeability in α7 nAChR deficient mice [8]. Endothelial cells may account for this effect [27]. However, since all α7 nAChR antibodies tested so far produce immunohistochemical labeling also in organs taken from α7 nAChR deficient mice [20,28], immunohistochemistry alone cannot decipher the cell-type specific α7-subunit distribution in the lung, and this issue remains to be solved.

Instead of α7 we observed expression of nAChR subunits α9, α10, β1, and β2, and to a variable extent α2, α3, α5, in rat AM. Mouse AM expressed nAChR subunits α9, α10, β2, and β4. To form classical, ion-conducting nAChR, α subunits combine as heteropentamers with β subunits or build α heteropentamers of α9α10 and homopentamers of α7 and α9 (for review, see [29]). The subunits detected in AM in the present study would allow combining the following nAChR pentamers: α3β2, α3α5β2, α9α10, and α9 as homopentamer. Since there is a constant expression of subunits α9 and α10 in AM, this combination as homo- or heteropentamer seems to be most likely, if pentamer formation occurs at all.

These subunits have been best characterized in the inner ear, where they form Ca^2+^-permeable ion channels involved in efferent modulation of hair cell function [30,31]. Our whole-cell patch clamp recordings and [Ca^2+^]_i _measurements in rat AM, however, neither revealed changes in membrane current in response to ACh nor in [Ca^2+^]_i _in response to nicotine. Similarly, a subpopulation of human T-lymphocytes expresses α9 and α10 nAChR subunits but fails to show transmembrane currents triggered by ACh [5], and nicotine does not cause alteration of [Ca^2+^]_i _in the rat AM cell line NR8383 [32] and in rat intravascular mononuclear leukocytes obtained from isogenic kidney transplants [7]. Thus, α9α10 nAChR subunits apparently do not form classical ionotropic receptors in cells of the immune system. Still, α9α10 nAChR subunits confer intracellular effects as our data demonstrate an acute α-bungarotoxin sensitive modulatory effect of nicotine upon ATP-induced calcium release from intracellular stores. In general, although to a much smaller extent than in rat cells, this effect was also present in macrophages isolated from C57BL6N and α7 nAChR subunit deficient mice, demonstrating its independency from the α7 nAChR subunit. Similarly, we recently identified a methyllycaconitine sensitive modulatory effect of nicotine upon ATP-induced rise in [Ca^2+^]_i _in rat mononuclear leukocytes obtained by vascular perfusion of isogenic kidney transplants [7]. In line with this observation, α9 subunit containing nAChR in outer hair cells of the inner ear do not exclusively assemble into ionotropic receptors, but form metabotropic receptors as well. Here, ACh also reduces ATP-induced rise in [Ca^2+^]_i _at a concentration that alone is insufficient to impact [Ca^2+^]_i_, and again this effect is α-bungarotoxin sensitive [33].

Atypical, non-ionotropic effects have also been reported for the nAChR α7 subunit. In T cells, this subunit fails to form a ligand-gated Ca^2+ ^channel but interacts with CD3ζ to modulate TCR/CD3 function [6]. Notably, α7 subunits in this complex exhibit a different agonist/antagonists profile than neuronal ionotropic α7 nAChR. Methyllycaconitine and α-bungarotoxin, both potent inhibitors of ionotropic α7 nAChR, indeed are strong agonists at T cells expressing nAChR α7 subunits [6]. Correspondingly, epibatidine, a highly potent agonist at ionotropic nAChR, failed to mimic the nicotine effect in our present experiments on rat AM.

In contrast to the well-characterized channel properties of nAChR, the mechanisms of atypical nAChR signaling are currently only poorly understood. In peritoneal macrophages, coupling of α7 nAChR to the Jak2-STAT3 signaling pathway resulting in STAT3 phosphorylation has been reported [3] which we could not observe in rat AM predominantly expressing α9 and α10 subunits. Membrane bound nAChR subunits have been demonstrated to interact with and to modulate signaling by β-arrestin [34], phosphatidyl-inositol-3-kinase [35], CD3ζ [6], and purinergic P2X-receptors [36,37]. The latter are involved in ATP-induced increase in [Ca^2+^]_i _by extracellular influx in human AM, since initial Ca^2+ ^transients are reduced by 40% in Ca^2+^-free medium [38]. In our present study of rat AM, however, the ATP-induced initial increase in [Ca^2+^]_i _and the modulatory effect of nicotine persisted in Ca^2+^-free solution, demonstrating interference of atypical nAChR with P2Y-receptor mediated Ca^2+^-release from intracellular stores. In support, we observed expression of P2Y purinergic receptors on AM, among them P2Y_2 _that mediates Ca^2+^-release from the endoplasmatic reticulum in mouse macrophages [11].

Extracellular ATP is well recognized as a "danger" or "host tissue damage" signal and is mostly regarded to promote inflammation [39,40]. In human AM, it couples to [Ca^2+^]_i _increases and stimulates IL-1β and IL-6 release albeit suppressing TNFα production [38]. In the rat AM cell line NR8383, ATP induces P2Y_2_- and Ca^2+^-dependent increase in CCL2 synthesis and release [41]. The CCL2-CCR2 axis is a crucial regulator of inflammatory cell influx into the murine lung [42,43]. Hence, the presently observed nicotinic attenuation of ATP-induced rise in [Ca^2+^]_i _can be considered as an anti-inflammatory mechanism triggered by atypical nAChR.

## Conclusions

Rat AM are equipped with modulatory nAChR with properties distinct from ionotropic nAChR mediating synaptic transmission in the nervous system. Their stimulation with nicotine dampens ATP-induced Ca^2+^-release from intracellular stores. Thus, the present study identifies the first acute receptor-mediated but atypical nicotinic effect on AM with anti-inflammatory potential.

## Competing interests

The authors declare that they have no competing interests.

## Authors' contributions

ZM, PH, GI and ZZ carried out the experimental work and drafted the manuscript. KSL and UP helped with the RT-PCR and immunofluorescence experiments. HK provided antibodies to α9 nAChR subunit. WC, JL, VG, MF participated in the experimental design and in manuscript preparation. WK initiated the study, designed the experiments, and participated in the manuscript preparation. All authors read and approved the final version of the manuscript.
